# Interventions that support the creation of dementia friendly environments in health care: protocol for a realist review

**DOI:** 10.1186/s13643-015-0168-2

**Published:** 2015-12-14

**Authors:** Melanie Handley, Frances Bunn, Claire Goodman

**Affiliations:** Centre for Research in Primary and Community Care, University of Hertfordshire, College Lane, Hatfield, Hertfordshire AL10 9AB UK

**Keywords:** Dementia, Health care, Dementia friendly health care, Hospitals, Patient outcomes, Realist review

## Abstract

**Background:**

Improving health-care outcomes for people living with dementia when they are admitted to hospital is a policy priority. Dementia friendly interventions in health care promote inclusion of patients and carers in decision-making and adapt practices and environments to be appropriate to the needs of people with cognitive impairment. While there has been a wealth of activity, the number of studies evaluating interventions is limited, and the majority focuses on reporting staff and organisational outcomes. By focusing on patient and carer outcomes, this review will aim to develop an explanatory account of how and in what circumstances dementia friendly environments in health care work for people living with dementia and with what outcomes.

**Method/design:**

Realist review is a theory-driven method which seeks to produce explanatory accounts of why interventions work and specifically, what combination of components are most effective in producing particular outcomes. Stakeholder interviews, a review of the literature, and an expert steering group workshop will be used to explore the assumptions behind interventions that are designed to enhance health care for people living with dementia to understand the underlying programme theories. The review will focus on studies that report patient and carer outcomes, including involvement in decision-making, length of stay and referral to long-term care, adverse incidents (e.g. patient distress, delirium falls, nutrition and hydration and infection), antipsychotic medication prescribing, evidence of patient-centred care and patient and carer satisfaction.

**Discussion:**

The review will provide an explanatory model about how dementia friendly interventions in hospital settings improve outcomes for people living with dementia and their family carers and in what circumstances for future testing and evaluation of future dementia friendly initiatives.

**Systematic review registration:**

PROSPERO CRD42015017562

## Background

It is estimated that at any one time a quarter of acute hospital beds are occupied by people living with a dementia, although their reason for admission may not be related to their dementia [1]. Comorbidities for people living with dementia are common and require appropriate attention from a range of health-care services [2, 3]. Inequalities in health-care outcomes for people living with dementia, such as lower use of analgesic medication during hospital admissions and reduced functional ability after discharge, have been widely reported, and it is acknowledged that these inequalities are detrimental to their health and quality of life [3–6]. A lack of leadership for dementia in secondary care [1], knowledge and training gaps for dementia in health-care staff [7, 8], the use of care practices which do not compensate for the effects of cognitive impairments [9], stigma and discrimination [10, 11] and environments which are disorientating [12] have all been identified as contributing to poorer health-care outcomes for people living with dementia. A number of initiatives have been developed and implemented in secondary health-care settings to address these areas with the aim of creating dementia-friendly health-care environments.

### Dementia friendly

The concept of ‘dementia friendly’ has been used to describe initiatives aimed at increasing the inclusion of people living with dementia in daily life and raising awareness of the issues they face among the wider population [13]. In its application to health-care settings, the concept of dementia friendly aims for the care and treatment of patients to be appropriate to their needs and of an equivalent standard expected by any patient [14]. Dementia friendly health care promotes inclusion of the person living with dementia and their carer in care and treatment discussions and decisions, with the aim of increasing positive outcomes for both [15]. In England, the Prime Minister’s dementia challenge [16] identified a number of areas for improvement in health care for people living with dementia and their carers. This included diagnosis rates, access to care, treatment support and information, coordination of care, admission and readmission to hospital, admissions to care homes and post-diagnosis support. Fundamental to addressing these challenges are multi-component interventions that educate staff in dementia awareness and care, improve health-care environments and increase access to relevant services ensuring people living with dementia and their carers are supported throughout the course of their condition (see Table 1).

**Table 1:** Dementia friendly health-care environments: anticipated outcomes and ways they might be achieved

Outcomes^a^	Achieved through
Skilled staff with time to care	• Staff training focused on dementia awareness
	• Support and reinforcement of skills in dementia care from clinical dementia leads
	• Identifying dementia champions as a resource for staff
	• Attention to staffing levels and staff mix
Partnership working with carers	• Assessment and communication tools that include carer knowledge and opinion
	• Flexible visiting hours for carers
	• Assessment of carers’ needs
Assessment and early identification	• Use of incentives for staff to complete training identify people in need of assessment
	• Screening and assessment tools
	• Protocols and pathways
	• Clinical reviews of medication
Individualised care (person-centred care)	• Assessment and documentation that focuses on the person’s biography, preferences and priorities and retained abilities
	• Activities to support social engagement and inclusion in everyday activities
	• Access to dementia and palliative care specialists who can guide staff in the provision of good quality care
Environments that are dementia friendly	• Environments that promote independence by being safe to walk around and navigate
	• Environments that are not confusing to a person living with dementia (e.g. shiny floors can be perceived as water, use of patterns and colour contrast)
	• Limiting ward moves during admission to minimise distress

^a^Based on [15]

### Evidence on problems experienced by people living with dementia and their carers and interventions

People living with dementia on entering hospital are at greater risk of adverse events, such as falls, poor nutrition and hydration, infections and delirium. If these occur during a hospital admission, they are likely to impact on the length of stay and may result in reduced function for the person [17–19]. Studies indicate people living with dementia admitted to hospital will stay at least an additional 4 days when compared with patients admitted for similar reasons and with similar profiles who do not have dementia [4, 20, 21].

It is also acknowledged that people living with dementia experience exclusion from decisions about their care and treatment [22]. A dementia friendly health-care environment, ideally, promotes and supports decision-making by people living with dementia and, as part of that process, involves their carers [23, 24]. Strategies that address inclusion include communication skills training for staff, the use of tools which document the preferences of the patient with dementia and practices that encourage partnership working between health-care professionals and family carers [25–27].

Few studies have evaluated interventions to improve health care for people living with dementia and their carers [28]. Evaluations have mostly focus on staff education, adaptions to models of service delivery and environmental changes to acute settings [29–33]. Primarily, they have reported staff and organisational outcomes, such as improved staff confidence and knowledge of dementia, economic costs, length of admission, readmission rates and place of discharge, with patient and carer outcomes more rarely operationalised or reported.

Approaches or interventions that show promise but have not been empirically tested to assess impact on patient outcomes are those which aim to improve communication between clinical staff and the carer (such as Alzheimer’s Society’s *This is me* booklet), the introduction of activities coordinators on hospital in-patient wards, and the use of dementia champions as change agents [8, 34, 35]. These are rarely used in isolation and are often adapted to meet the needs of the local context, meaning interventions are multi-component and context sensitive. Understanding the components of interventions that support patient outcomes, such as reduced distress, increased recovery, participation in care and the promotion of independence, can help to develop an explanatory account of what works in what circumstances.

Realist approaches are theory-driven and recognise that interventions themselves are not the cause of change; it is the resources that the intervention provides and reaction to those resources by the people that use them that generates change [36]. The effectiveness of programmes to address the known problems of being a patient with dementia is recognised as contingent not only on specific training (for example) in being dementia aware but also on ‘contextually situated decision-making’ [37].

### Aims and objectives

The overall aim is to identify features or mechanisms of programmes and approaches that aim to make health-care delivery in secondary health-care settings more dementia friendly, provide a context-relevant understanding of the mechanisms by which interventions achieve different outcomes for people living with dementia and their family carers and make explicit the barriers and facilitators to implementation. Specifically, we will

1. Identify how dementia friendly interventions are thought to achieve the desired patient and carer outcomes
2. Identify the perceived enablers and inhibiters for the creation of dementia friendly health-care environments
3. Identify what is it about dementia friendly health-care interventions that works for people living with dementia and their carers, in what circumstances and why.

### Methodological approach

This review draws on the assumptions of realist theory [38, 39] and linked ideas of critical realism [40]. Interventions implemented into health care rely on human agency to effect change. The realist approach suggests that the resources provided by the intervention and the context it is implemented have the ability to produce a limited number of potential responses to the intervention which will impact on the outcomes. A knowable, independent reality will constrain the way an individual reacts to an intervention, whether they are aware of these influences or not [41]. It is important to understand these phenomena when seeking to explain why an intervention has worked or not. Interventions in health care are invariably multi-component and complex in design allowing for a spectrum of outcomes to occur under different conditions, from successful to unsuccessful implementation. By focusing on the building, testing and refinement of theory, realist review approaches are able to incorporate the diversity of outcomes to provide an explanatory account of the key features which enable or inhibit the effectiveness of an intervention [36].

Realist review assumes evidence that is relevant and is available from diverse sources, offering a way of synthesising different literature including policy documents, organisational presentations, empirical evidence and editorials, and primary studies which utilise a variety of methods for evaluating complex social interventions [42–44]. It is an iterative process that builds and refines theory throughout the process. This review will follow RAMESES standards [39, 45].

## Methods/design

Interviews with stakeholders, a review of the literature and an expert steering group workshop will be used to explore theoretical assumptions about why and how interventions that promote dementia friendly health-care work (or not) in secondary health-care settings, how they work with different populations and what are the significant mechanisms for change. There are a number of ways to conceptualise the development of dementia friendly environments in health care, and it is likely that the review will be informed by theoretical work on the following:

- Human rights and social model of disability framework [46–50] that are focused on the adaption of current models of care in order to promote inclusion and engagement
- Organisational theories of change perspectives [51, 52] that focus on the way that values and beliefs defined at a strategic level are embedded across the workforce in order to appropriately meet the needs of people living with dementia and improve the efficiency of services
- Perceptions of the roles clinicians, patients and carers hold [53] and approaches that contribute to breaking down boundaries and barriers to promote shared decision-making for the person living with dementia and their carer, where their insights of living with the condition are valued [54].
- Educational theories for change where increasing the awareness of dementia in the health-care workforce will lead to a critical consciousness that can be further harnessed to transform services [55, 56].

### Stages of the realist review

There are three overlapping and iterative phases to this realist review (Table 2). These phases do not necessarily follow a linear format. Sources are identified and revisited, new evidence can be incorporated and inclusion criteria can be expanded to include transferable learning throughout the process in order to develop credible theories for how and why an intervention works.

**Table 2:** Phases of realist review

Phase 1
• Defining the scope of the review: concept mining and theory development
◦ Interviews with stakeholders
◦ Scoping the literature
◦ Mapping key programme theories
◦ Prioritising key programme theories
Phase 2
• Search for primary studies
◦ Defining search terms
◦ Search strategy
◦ Study screening
• Selection and appraisal of documents
◦ Inclusion and exclusion criteria
• Data extraction
Phase 3
• Analysis and synthesis
• Dissemination
◦ Expert steering group
◦ Additional dissemination methods

### Phase 1

#### Defining the scope of the review: concept mining and theory development

In phase 1, we will undertake a preliminary scoping of a selection of key literature (e.g. relevant descriptions and evaluations of dementia friendly initiatives in health-care settings). We will map the range of international health-care-based interventions, explore their underlying rationale, detail the resources, such as training or communication tools the intervention provides and explore how the interventions are supposed to work. This will inform the identification of context-mechanism-outcome configurations that inform the articulation of prominent programme theories and help to refine the search strategy for the detailed review in phase 2.

Key word searches will be performed using PubMed and CINHAL to identify the broad spectrum of work. Google Scholar will be used to identify grey literature. As initiatives may predate recent policy documents (such as the National Dementia Strategy [57], the Prime Minister’s dementia challenge [16] and the Alzheimer’s Society’s dementia friendly communities [13]), search terms will be chosen to reflect the variety of ways in which ‘dementia friendly’ initiatives might be interpreted or implemented. Potential terms include the following:

‘dementia friendly’

‘dementia friendly AND health care’

‘dementia appropriate AND health care’

‘dementia awareness AND health care’

‘dementia person-centred care AND health care’

‘dementia champions’

‘dementia AND liaison’

‘dementia AND ward’

‘dementia education’

‘dementia training’

‘dementia nurse specialist’

‘dementia lead*’

Searches will be time limited to between 2000 and 2015 to reflect the impact of the work of Kitwood [58] in person-centred care. This has become the accepted best practice model of care for people living with dementia and has influenced dementia practice; moving away from a purely biomedical view to one that incorporates the effect of social factors. Searches will be restricted to English language. No other restrictions will apply.

In addition, in order to understand the underlying assumptions or theories that inform dementia friendly initiatives in health care and how effectiveness is defined, we will undertake semi-structured telephone or face-to-face interviews with up to 15 purposively sampled stakeholders. These will include commissioners, clinicians, academics with expertise in dementia care research and people living with dementia and their family carers. Stakeholder interviews will be conducted with a topic guide and, with permission, digitally recorded. Interviews will be analysed with framework analysis using the five steps identified by Ritchie and Spencer [59]: familiarisation, identifying a thematic framework, indexing, charting and mapping and interpretation.

Evidence from the literature will be coded and organised into if-then statements to develop a conceptual framework [60]. Data from the interviews and literature will be used to identify context-mechanism-outcome configurations which can be used to develop possible programme theories for testing in phase 2.

### Phase 2

#### Retrieval and review

The inclusion criteria will be refined in light of the emerging data and the theoretical development in phase 1 but are likely to include evidence sources that cover the following:

- People with mild, moderate or advanced dementia of any type, e.g. Alzheimer’s disease, vascular dementia, Lewy body dementia, Parkinson’s disease dementia, fronto-temporal dementia and alcohol-related dementia
- Studies of any intervention or initiative designed to make secondary health-care settings more ‘dementia friendly’. This might include those which promote the inclusion and engagement of people living with dementia and their family carers, which adapt care practices and/or the environment to reduce adverse incidents and promote independence, or which establish roles with the specific remit of improving outcomes for people living with dementia
- Studies that provide evidence on barriers and facilitators to the implementation and uptake of interventions designed to make healthcare environments in secondary care more dementia friendly
- Studies that offer opportunities for transferable learning. For example, studies in hospitals that aim to reduce health-care inequalities in other vulnerable groups, such as people with learning disabilities or mental health issues, or those outside of health care (e.g. Healthy Cities) if they have included older people living with dementia and are drawing on similar principles of engagement and delivery to achieve equivalent outcomes (e.g. inclusion and access).

##### Outcomes

The primary aim of dementia friendly health care is to improve the health and wellbeing of people living with dementia and their family carers and to ensure equity of access and treatment [14, 61]. This review, therefore, will only include studies that report patient and carer outcomes. These outcomes will be established by the project team as an iterative process but are likely to include the following: (1) patient and carer involvement in decision-making, (2) length of hospital stay, (3) occurrence of adverse incidents (e.g. falls, nutrition and hydration, infection and delirium), (4) use of antipsychotic medication, (5) needs assessment (for patient and carer), (6) patient and carer satisfaction and (7) access to care. These outcomes are not only important for maintaining health and function for people living with dementia but also for ensuring their choices and rights are respected and supported in an appropriate way.

##### Searching for relevant studies

Search terms from phase 1 will be extended to reflect the theories that emerge from the initial scoping of the literature and to ensure we capture the range of potential interventions and theories. If necessary, search terms will be broadened to include groups of patients other than people living with dementia. This would enable us to capture insights from literature pertaining to elements of the programme theory and to build a more refined understanding of the interacting features. While studies will not be limited to the UK, as the international literature will provide important evidence for theoretical understanding, they will be limited to those available in English language and which are likely to be relevant to UK systems of health care.

##### Search strategy

An example of the search terms for PubMed is given in Table 3. Search terms will be entered into the following electronic databases: Cochrane Library (including CENTRAL, CDSR, DARE, HTA), CINAHL, PubMed, NHS Evidence and Scopus.

**Table 3:** Search terms for phase 2

Following the scoping in phase 1, phase 2 search terms will be refined to reflect the theory under investigation. For example, an emerging theory about organisational processes in change management would generate search terms of
‘change agent’ ‘change management’ ‘knowledge translation’ ‘opinion leader’ ‘knowledge transfer’
In PubMed, these would be operationalised using Boolean terms:
hospital AND (change agent OR change management OR knowledge translation OR opinion leader OR knowledge transfer)

Additionally, databases from disciplines outside health care will be searched to reflect the dominant fields of the theory. For example, if the theory has a focus on organisational change, databases with a focus on education (e.g. Education Research Complete, ERIC) and human resources (e.g. XpertHR) will be searched.

The following extensive lateral search techniques will be used:

- Interrogating reference lists of relevant reviews and primary studies
- Snowballing (forward and backward citation tracking) [62]
- Key word searches in Google Scholar
- Searching of grey literature
- Searching the websites of charities, user groups and patient and carer associations, such as Alzheimer’s Society, Dementia Action Alliance, Age UK and Carers UK.

Searches will continue throughout the synthesis as realist review is an iterative process. Additional studies will be used to refine theory development until theoretical saturation has been achieved [63, 64].

##### Study screening and data extraction

Search results will be downloaded into bibliographic software and, where possible, duplicates deleted. Studies will initially be screened by title and abstract for relevance to the research questions by one reviewer (MH). Full texts of potentially relevant manuscripts will be screened for inclusion based on whether they demonstrate both relevance (whether the study has contributed to specific propositions relevant to the programme theory building and testing) and rigour (that the evidence used is of sufficient quality to help clarify the particular proposition it is being used to address) [36, 39]. These decisions will continue throughout the synthesis as their relative contribution to the programme theory is assessed throughout the refinement process. A random subset of papers will be screened by the second reviewers (CG and FB) to ensure that data identified within the documents are relevant, contributing to the appraisal and development of the programme theory. Where there is disagreement about inclusion, decisions will be made through ongoing discussion with debates, and resolutions being recorded and reported.

For studies that meet the test of relevance, data will be extracted by one review (MH) onto a specially designed data extraction form which will enable us to organise the theories of initiatives and patterns that emerge from different context, mechanisms, and outcome configurations. Strengths and weaknesses of the study will be highlighted, which will help inform tests of rigour. Study characteristics such as design, setting, participants and sample size will be included [39].

### Phase 3

#### Analysis and synthesis

After initial characteristics have been extracted, relevant text from the literature will be entered into NVivo and coded by theme by one reviewer (MH) to assist the refinement of the programme theories [65, 66]. Initial themes and codes will be shared with the team (MH, FB, CG) to reflect upon the emerging ideas and discuss the resonance of these. Data will be used to corroborate or contradict different parts of the theories, testing the ideas from the earlier stages of the review to build an evidence-based explanation of the relationships between context, mechanism and outcome. Triangulation will be used to adjudicate between and across the findings from studies, highlighting positive and negative instances. Programme theories will be discussed with the team (MH, FB, CG) and revised to reflect the emerging evidence. Justification for the amendments will be documented. The synthesis will result in a theoretical explanation of what it is about (specific) interventions designed to support the creation of dementia friendly environments in health care that works to improve patient outcomes, in what circumstance, how and why.

#### Dissemination

##### Expert steering group workshop

Findings from the full review will be discussed with an expert steering group in a day’s workshop. The objectives of the workshop are to check that the findings and recommendations from the review have relevance and resonance with the stakeholders and to highlight any possible alternative interpretations.

##### Participants

Stakeholders will be invited using the recruitment strategy employed for the interviews (i.e. identifying a purposive sample from discussions with colleagues, Internet searches and snowballing from other participants) with an emphasis of inviting people living with dementia and practitioners. The workshop will involve up to 20 stakeholders who represent a balanced mix of interests.

##### Consent

All stakeholders will be asked to provide written consent or, where this is not possible, witnessed verbal consent will be provided. All discussions will remain confidential to the group. Stakeholders will be asked to respect the views of other members and talk one at a time.

##### Data collection

Stakeholders will be asked to participate in activities during the workshop. These will focus on the findings of the review and whether these findings are recognised by the group. Points arising from these small group activities will be written up on flipchart paper by one member of the group or the facilitator and reported back to the whole group. The flipchart paper will be collected by the researcher at the end of the workshop to assist with further analysis. Additionally, the workshop will be recorded to assist with further analysis of the review. This recording will be transcribed, anonymised and entered into NVivo for analysis by one reviewer (MH).

### Ethics

For phase one interviews, ethical approval was secured from the University of Hertfordshire (HSK/PG/UH/00339). The study will not require NHS ethics approval. The review has been registered with PROSPERO (CRD42015017562).

## Discussion

People living with dementia admitted to hospital need health-care services that consider the impact of the patient’s cognitive impairment and adapt practices appropriately to provide a standard of care that is equitable to the expectations of other patients. From a range of published and other sources, the review will use a theory-driven evidence synthesis to provide testable causal inferences about what influences effective dementia-friendly interventions in hospital settings, including what enables or inhibits this process. This explanatory account of what it is that supports positive outcomes for people living with dementia and their carers during an admission to hospital will be the basis for future testing in different settings with the ultimate purpose of developing a framework that be used to develop and evaluate dementia-friendly initiatives.

## Abbreviations

CINHAL: Cumulative Index to Nursing and Allied Health Literature
RAMESES: Realist and Meta-Narrative Evidence Synthesis
